# SAMHD1 restricts HIV-1 infection in dendritic cells (DCs) by dNTP depletion, but its expression in DCs and primary CD4^+^ T-lymphocytes cannot be upregulated by interferons

**DOI:** 10.1186/1742-4690-9-105

**Published:** 2012-12-11

**Authors:** Corine St Gelais, Suresh de Silva, Sarah M Amie, Christopher M Coleman, Heather Hoy, Joseph A Hollenbaugh, Baek Kim, Li Wu

**Affiliations:** 1 Center for Retrovirus Research, Department of Veterinary Biosciences, The Ohio State University, 1900 Coffey Road, Columbus, Ohio, 43210, USA; 2 Department of Microbiology & Immunology, University of Rochester School of Medicine and Dentistry, 601 Elmwood Ave, Box 672, Rochester, New York, 14642, USA; 3 Department of Microbial Infection and Immunity, The Ohio State University, Columbus, Ohio, 43210, USA

**Keywords:** HIV-1 restriction, SAMHD1, Dendritic cells, Interferon, Intracellular dNTPs

## Abstract

**Background:**

SAMHD1 is an HIV-1 restriction factor in non-dividing monocytes, dendritic cells (DCs), macrophages, and resting CD4^+^ T-cells. Acting as a deoxynucleoside triphosphate (dNTP) triphosphohydrolase, SAMHD1 hydrolyzes dNTPs and restricts HIV-1 infection in macrophages and resting CD4^+^ T-cells by decreasing the intracellular dNTP pool. However, the intracellular dNTP pool in DCs and its regulation by SAMHD1 remain unclear. SAMHD1 has been reported as a type I interferon (IFN)-inducible protein, but whether type I IFNs upregulate SAMHD1 expression in primary DCs and CD4^+^ T-lymphocytes is unknown.

**Results:**

Here, we report that SAMHD1 significantly blocked single-cycle and replication-competent HIV-1 infection of DCs by decreasing the intracellular dNTP pool and thereby limiting the accumulation of HIV-1 late reverse transcription products. Type I IFN treatment did not upregulate endogenous SAMHD1 expression in primary DCs or CD4^+^ T-lymphocytes, but did in HEK 293T and HeLa cell lines. When SAMHD1 was over-expressed in these two cell lines to achieve higher levels than that in DCs, no HIV-1 restriction was observed despite partially reducing the intracellular dNTP pool.

**Conclusions:**

Our results suggest that SAMHD1-mediated reduction of the intracellular dNTP pool in DCs is a common mechanism of HIV-1 restriction in myeloid cells. Endogenous expression of SAMHD1 in primary DCs or CD4^+^ T-lymphocytes is not upregulated by type I IFNs.

## Background

Myeloid lineage cells such as monocytes, macrophages and dendritic cells (DCs) are important immune cells that elicit innate and adaptive immune responses to a variety of pathogens, including viruses. HIV-1 is known to replicate poorly in myeloid cells; however, these cells play an important role in promoting dissemination of HIV-1 to CD4^+^ T-lymphocytes, the major target of HIV-1 infection [1,2]. In contrast to HIV-1, HIV-2 and simian immunodeficiency virus (SIV) from the sooty mangaby lineage are able to efficiently infect myeloid lineage cells by a mechanism initially attributed to the Vpx protein mediating degradation of an unknown host cellular restriction factor [3]. Restriction factors are a group of cellular proteins that can block viral replication in cells and are typically upregulated by type I interferons (IFNs) [4-6]. Well-characterized HIV-1 restriction factors include apolipoprotein B mRNA editing enzyme, catalytic polypeptide-like 3G (APOBEC3G) [7], tripartite motif 5α (TRIM5α) [8], and tetherin (also known as BST-2 or CD317) [9,10].

SAM domain and HD domain-containing protein 1 (SAMHD1) was initially identified in myeloid cells as an HIV-1 restriction factor that was degraded by HIV-2/SIV Vpx [11-13]. Recent studies also revealed that SAMHD1 restricts HIV-1 infection in resting CD4^+^ T-cells [14,15]. Vpx-mediated degradation of SAMHD1 was found to relieve HIV-1 restriction in myeloid cells and resting CD4^+^ T-cells, allowing enhancement of HIV-1 infection [11-18]. SAMHD1 is a dGTP-regulated deoxynucleoside triphosphate (dNTP) triphosphohydrolase that hydrolyzes dNTPs *in vitro*[19,20]. SAMHD1-mediated HIV-1 restriction occurs via endogenous SAMHD1 depleting the intracellular dNTP pool, thereby inhibiting HIV-1 reverse transcription and viral infection [14,16,18,21]. However, the intracellular dNTP pool concentration in DCs has not been reported. Moreover, the effect of SAMHD1 on regulating the dNTP pool in DCs remains to be investigated.

Mutations in *SAMHD1* are associated with a rare genetic disorder known as Aicardi-Goutieres syndrome (AGS) with symptoms of a congenital viral infection likely due to excessive production of IFN-alpha (IFNα) and increased immune activation [22,23], suggesting that SAMHD1 may act as a negative regulator of the type I IFN response. SAMHD1-deficient CD14^+^ monocytes and resting CD4^+^ T-lymphocytes from AGS patients are highly susceptible to HIV-1 infection *in vitro*[13-15], suggesting that SAMHD1 may be critical for inhibiting HIV-1 infection *in vivo*. Moreover, IFNα treatment of monocytes isolated from a healthy donor up-regulated the levels of SAMHD1 protein [13], indicating that SAMHD1 is type I IFN-inducible in monocytes. However, it remained unknown whether endogenous SAMHD1 expression in DCs and primary CD4^+^ T-cells can be upregulated by type I IFNs.

In this study, we sought to understand the mechanism of SAMHD1-mediated HIV-1 restriction by characterizing SAMHD1 protein expression and response to IFNs in both DCs and primary CD4^+^ T-lymphocytes. We show that Vpx-mediated degradation of SAMHD1 in DCs significantly enhances HIV-1 infection and accumulation of late reverse transcription products, and increases the intracellular dNTP pool. We observed that endogenous SAMHD1 in primary DCs or CD4^+^ T-lymphocytes are not readily upregulated by type I IFNs. Our results provide new information on the cellular mechanism of SAMHD1-mediated HIV-1 restriction in DCs and the function of SAMHD1 as an HIV-1 restriction factor.

## Results

### Vpx-mediated SAMHD1 degradation in DCs significantly increases HIV-1 infection and accumulation of late reverse-transcription products

Treatment of DCs, monocytes, macrophages, and differentiated monocytic cell lines with Vpx-containing SIV virus-like particles (VLPs) has been shown to enhance HIV-1 infection in these cells [11-13,16,24-30]. Vpx-mediated degradation of SAMHD1 accounts for the enhanced HIV-1 infection in myeloid cells [11-13,16]. We confirmed that Vpx-mediated degradation of SAMHD1 enhanced both single-cycle and replication-competent HIV-1 infection in DCs. DCs were transduced with either Vpx-negative SIV VLPs or VLPs containing Vpx from HIV-2 or SIV. These VLPs lack viral genetic material and can be used to effectively deliver Vpx protein to DCs [3,31]. Mock-transduced DCs served as an additional negative control to exclude any effect of other SIV proteins on SAMHD1 expression. VLP-transduced DCs were infected with single-cycle or replication-competent HIV-1, and viral infection was measured by detecting luciferase activity and p24 capsid release, respectively.

**Figure 1:**
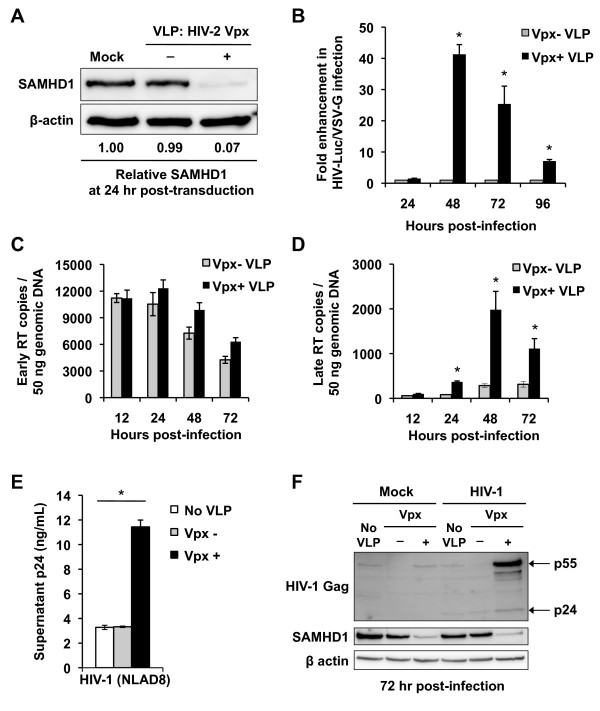
**Vpx-mediated SAMHD1 degradation in DCs efficiently increases HIV-1 infection and accumulation of late reverse-transcription products.** Monocyte-derived DCs were transduced with SIV VLPs containing HIV-2 Vpx (Vpx +) or not (Vpx -). Mock transduction (no VLPs) was used as a negative control. (**A**) Whole cell lysates were subjected to immunoblotting for SAMHD1 at 24 hr post-transduction. β-actin was used as a loading control. Relative levels of SAMHD1 compared to β-actin are shown. (**B**) VLP-transduced DCs were infected with HIV-Luc/VSV-G at a multiplicity of infection (MOI) of 1 and the infection was detected by measuring luciferase activity in the cell lysates at the indicated times post-infection. Fold enhancement of HIV-1 infection (VLP without Vpx control set to 1) is shown. (**C**) HIV-1 early reverse-transcription products (early RT) in DCs transduced with VLPs. The early reverse-transcription copies were measured by qPCR at the indicated times post-HIV-1 infection (MOI of 1). (**D**) Increased HIV-1 late reverse-transcription products (late RT) in DCs transduced with VLPs. The late reverse-transcription copies were measured by qPCR at the indicated times post-HIV-1 infection (MOI of 1). (**E** and **F**) VLP-transduced DCs were infected with replication-competent, R5-tropic, HIV-1_NLAD8_ (MOI of 0.5). At 3 days post-infection, HIV-1 p24 in the supernatant was measured by ELISA. (**F**) Expression of SAMHD1, HIV-1 Gag (p55 and p24), and β-actin in NLAD8 HIV-1 infected DCs was detected by immunoblotting. The data shown represent one of three independent experiments. Error bars represent standard deviation of the mean of duplicate samples. (**B**, **D**, and **E**) The asterisks indicate a significant difference (*p* <0.05) compared with the controls of no VLP and/or (Vpx -).

Vpx efficiently degraded endogenous SAMHD1 in DCs at 24 hr post-transduction (Figure 1A) and significantly enhanced vesicular stomatitis virus G protein (VSV-G)-pseudotyped single-cycle HIV-1 infection of DCs by 1.5- to 41-fold over a period of 24 to 96 hr post-infection (*p* <0.05), relative to negative controls (Figure 1B). The increase in HIV-1 infection of myeloid cells in the presence of Vpx-mediated SAMHD1 degradation has been attributed to increased production of late HIV-1 reverse transcription products, which constitute full-length viral cDNA [11,12,16]. To better understand the mechanism underlying SAMHD1-mediated HIV-1 restriction in DCs, we quantified the products of HIV-1 early and late reverse transcription over a time course ranging from 12 to 72 hr post-infection in the presence or absence of Vpx. This time course is based on our previous kinetics studies of HIV-1 infection and viral cDNA measurement in DCs [32-36]. We found that DCs transduced with Vpx(+) VLPs and infected with HIV-1 had no significant difference in the production of early minus strand reverse transcription products at 12 to 72 hr post-infection (Figure 1C). However, analysis of late reverse transcription products revealed 4.5-, 6.9-, and 3.6-fold increases at 24, 48, and 72 hr post-infection (*p* ≤0.001), respectively, in the presence of Vpx (Figure 1D). These results are in agreement with a previous study of non-dividing macrophages [12], suggesting that the synthesis of HIV-1 early reverse transcripts is less restricted by SAMHD1 relative to late reverse transcripts in myeloid cells.

Furthermore, we observed that Vpx-mediated SAMHD1 degradation increased infection of DCs with R5-tropic, replication-competent HIV-1_NLAD8,_ as evidenced by 4-fold enhancement of p24 production in the supernatants from infected DCs at 72 hr post-infection (*p* <0.001) (Figure 1E). Immunoblotting of DC lysates from the same experiment indicated that *de novo* production of HIV-1 Gag p55 was significantly enhanced in SAMHD1 knockdown cells compared to negative controls (Figure 1F), confirming productive infection. Similar results were obtained using VLPs containing endogenous SIVmac251 Vpx (Vpx is expressed from the SIVmac251 proviral DNA rather than *trans*-complemented) (data not shown). These results demonstrate that Vpx-mediated SAMHD1 degradation efficiently enhances HIV-1 infection of DCs and demonstrates that knockdown of SAMHD1 in DCs enables accumulation of full-length viral cDNA.

### Vpx(+) VLP treatment increases the intracellular pool of dNTPs in DCs

The intracellular dNTP concentrations of DCs have not been previously published, and it is not known if SAMHD1 modulates the dNTP pool in primary DCs. To address these questions, we determined the intracellular dNTP levels of DCs and quantified the effect of Vpx treatment on the dNTP pool in DCs using a single nucleotide incorporation assay. DCs from two independent donors were treated with SIV VLPs (+/−) Vpx. Cell samples were harvested at 0, 12, and 24 hr post-VLP treatment and intracellular dNTP levels were determined.

**Figure 2:**
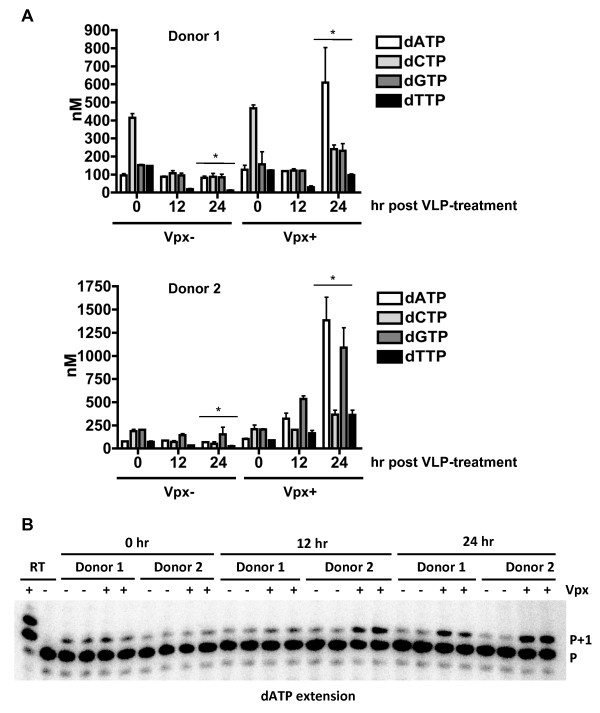
**Vpx**-**treatment increases the intracellular dNTP pool in DCs.** Monocyte-derived DCs treated with SIVmac VLPs with and without Vpx were collected at 0, 12, and 24 hours post VLP treatment. (**A**) The intracellular dNTP pool of VLP-transduced DCs was measured using the single nucleotide incorporation assay. The data show results of DCs from two independent healthy donors. Error bars represent standard deviation of the mean of duplicate samples. The asterisks indicate a significant difference (*p* <0.001) compared with the (Vpx -) controls at 24 hr post VLP-transduction. (**B**). A representative HIV-1 RT-based single nucleotide primer extension gel is shown for dATP extension for both donors. Reactions (+/−) RT were used to indicate unextended primer (P) versus and extended primer (P+1). Vpx(+) VLP treated samples at 24 hr were diluted 1:3 to prevent complete primer extension and maintain results within the linear range for quantification purposes.

Vpx(−) VLP treated DCs, which served as a negative control, maintained overall dNTP levels below 203 nM during the time course, except DCs from donor 1 displayed an elevated level of dCTP (415 nM) at time 0 post-treatment (Figure 2A). These results indicate the variability of dNTP concentrations among DCs from different donors. However, compared to Vpx(−) VLP-transduced DCs, when DCs were treated with Vpx(+) VLPs to degrade SAMHD1, an increase in the dNTP levels was evident, with dATP, dCTP, dGTP, and dTTP levels increasing significantly (7-, 3-, 3, and 9-fold, respectively; *p* <0.001) at 24 hr post-treatment in donor 1 (Figure 2A). Compared to Vpx(−) VLP-transduced DCs, Vpx(+) VLP-treated DCs from donor 2 also showed increased dNTP levels at 24 hr post-transduction (20-, 7-, 7- and 14-fold for dATP, dCTP, dGTP, and dTTP, respectively; *p* <0.001) (Figure 2A). Overall, Vpx treatment of DCs from two donors increased the intracellular dNTP pool 1.1- to 4.9- fold and 3- to 20-fold at 12 hr and 24 hr post-treatment, respectively (Additional file 1: Table S1).

A representative gel image of HIV-1 RT-based single nucleotide primer extension assay is shown for dATP extension (Figure 2B). Extended primer (P+1) can be visualized with increasing intensity in both donor 1 and donor 2 samples in the presence of Vpx at 12 and 24 hr post VLP treatment. These data suggest that primary DCs possess low levels of intracellular dNTPs, in keeping with other non-dividing primary cells, such as macrophages and resting CD4^+^ T-lymphocytes [14,21,37-39]. Our results also suggest that SAMHD1 plays an important role in maintaining the intracellular dNTP pool in DCs.

### Type I and type II IFN treatment does not upregulate SAMHD1 protein expression in DCs

**Figure 3:**
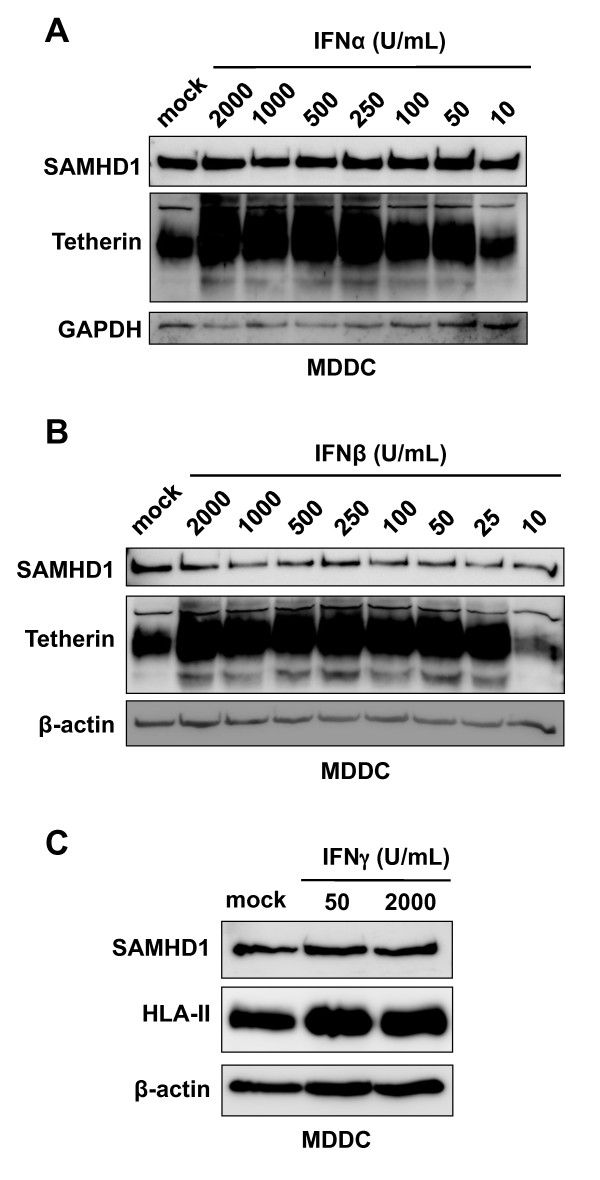
**Type I and type II IFN treatment does not upregulate SAMHD1 expression in DCs.** (**A** and **B**) Monocyte-derived DCs (MDDCs) were treated with different amounts of IFNα or IFNβ as indicated for 24 hr and whole cell lysates were subjected to immunoblotting for SAMHD1 and tetherin expression. GAPDH or β-actin was used as a loading control. (**C**) MDDCs were mock treated or treated with IFNγ at the indicated concentrations for 24 hours. Whole cell lysates of treated MDDCs were subjected to immunoblotting for SAMHD1 or HLA-II. β-actin was used as a loading control. The MDDC data shown represents one of two independent experiments using cells from different donors.

SAMHD1 was initially identified as a homologue of the mouse IFN-gamma (IFNγ)-inducible protein [40]. Consistent with other HIV-1 restriction factors, SAMHD1 is induced by type I IFN treatment in primary monocytes [13]. To investigate the effect of type I IFNs on SAMHD1 protein expression in DCs, we treated DCs with increasing amounts of either IFNα or IFN-beta (IFNβ) for 24 hr and then detected endogenous SAMHD1 protein expression. Surprisingly, immunoblotting for SAMHD1 revealed that there was no major change in the protein levels of SAMHD1 upon treatment with either IFNα (Figure 3A) or IFNβ (Figure 3B). To confirm that the IFN treatment was effective, we detected tetherin expression, in type I IFN-treated DCs (Figure 3A and 3B, middle panels) as observed previously [32]. We also found that IFNγ (a type II IFN) did not affect SAMHD1 expression in DCs (Figure 3C). The positive control for IFNγ stimulation (human leukocyte antigen class II, HLA-II) confirmed the effectiveness of IFNγ treatment (Figure 3C, middle panel). Overall, these results demonstrate that endogenous expression of SAMHD1 protein in DCs is further upregulated by treatment with type I or type II IFNs.

### Type I and type II IFN treatment causes a transient increase in SAMHD1 mRNA levels at early time points in primary DCs

**Figure 4:**
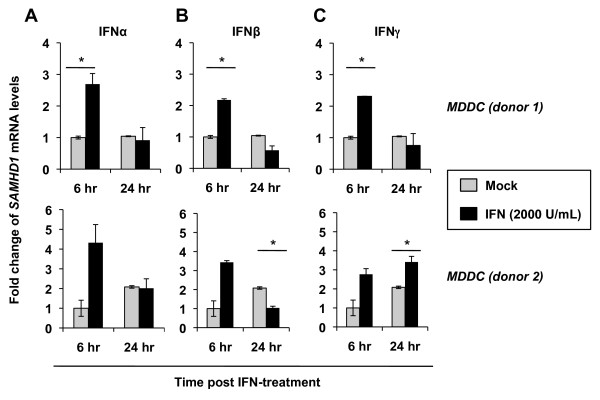
**Type I and type II IFN treatment upregulates *SAMHD1 *mRNA levels in DCs at 6 hr post**-**treatment.** Monocyte-derived DCs (MDDC) from two independent donors were either mock treated, or treated with 2000 U/mL of (**A**) IFNα, (**B**) IFNβ or (**C**) IFNγ as indicated for either 6 or 24 hr. Cell pellets were collected and total RNA was extracted and 200 ng of the total RNA were used for cDNA synthesis. Quantitative PCR was performed using *SAMHD1* cDNA specific primers and all data was normalized to *GAPDH*. Data shown represent fold change in mRNA levels compared to 6 hr mock treated cells. The asterisks indicate a significant difference (*p* <0.05) compared with the mock controls.

To examine whether IFN treatment of DCs affected *SAMHD1* mRNA levels, we treated DCs from two donors with 2000 U/mL of IFNα, IFNβ or IFNγ for either 6 or 24 hr and performed quantitative PCR analysis. We observed that at 6 hr post-treatment with all three types of IFNs, levels of SAMHD1 mRNA increased 2.1- to 2.7-fold (IFNα, *p*=0.0038; IFNβ, *p* = 0.0025; IFNγ, *p* = 0.001) above mock treated DCs (Figure 4A-C, donor 1). By 24 hr post-treatment, SAMHD1 mRNA levels regressed to levels comparable to or slightly below mock treated DCs (Figure 4A-C). Similar trends were observed for a second donor although the fold change in mRNA levels at 6 hr post-treatment compared to mock treated cells ranged from 2.8- to 4.3-fold (Figure 4A-C, donor 2). These data indicate that *SAMHD1* mRNA in DCs can be transiently upregulated by IFNs for a short period post-treatment; however, it does not translate to an increase in SAMHD1 protein expression.

### Kinetics analysis of SAMHD1 protein and mRNA in DCs treated with IFNα

**Figure 5:**
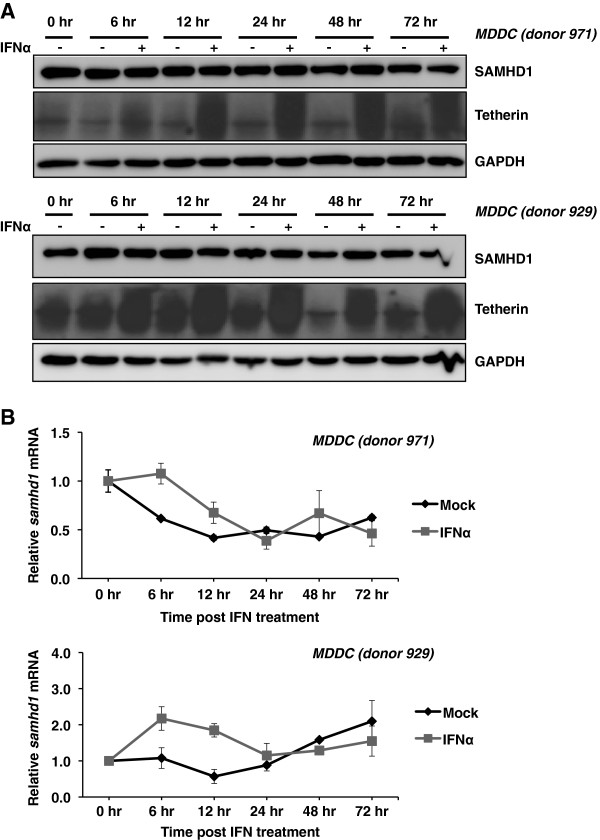
**Kinetics analysis of SAMHD1 protein and mRNA in DCs treated with IFNα.** Monocyte-derived DCs (MDDC) from two independent donors were mock treated, indicated with “-”, or treated with 2000 U/mL IFNα, indicated with “+”, for the indicated times. (**A**) Whole cell lysates were subjected to immunoblotting for SAMHD1 and tetherin expression. GAPDH was used as a loading control. (**B**) Quantitative PCR was performed using *SAMHD1* cDNA specific primers and all data was normalized to *GAPDH*. Data shown represents fold change in mRNA levels compared to 0 hr mock treated cells.

As we observed that at 24 hr post-IFN treatment, SAMHD1 protein levels were not increased and that mRNA levels were only increased transiently at 6 hr post-IFN treatment, we thus examined the steady state levels of SAMHD1 protein and mRNA over a more extensive time course from 6 to 72 hr post-IFNα treatment. We treated DCs from two donors with IFNα and performed immunoblotting for SAMHD1 protein and qPCR analysis for *SAMHD1* mRNA detection. Over the time period of 6 to 72 hr post-IFNα treatment, SAMHD1 protein levels in the mock treated samples did not vary; indicating that SAMHD1 is stably expressed in DCs (Figure 5A). Analysis of SAMHD1 protein in the IFNα-treated samples also showed no change in protein levels across the time course (Figure 5A), confirming that SAMHD1 protein levels are not upregulated by IFNα treatment. The effectiveness of IFNα treatment was confirmed by up-regulation of tetherin protein levels observed from 6 to 72 hr post-treatment in DCs from both donors (Figure 5A). Analysis of *SAMHD1* mRNA levels in DCs from both donors indicated small variations in mRNA levels in mock-treated cells throughout the time course. In IFNα-treated DCs, minor increases in mRNA levels were observed at 6 and 12 hr post-treatment (donor 971, 1.7- and 1.6-fold respectively; donor 929, 2- and 3.3-fold respectively; Figure 5B). By 24 hr post-treatment, *SAMHD1* mRNA levels returned to levels comparable with mock treated cells (Figure 5B). These results confirm that *SAMHD1* mRNA levels in DCs can be transiently induced by treatment with IFNα for 6–12 hr, and that there is no effect of IFNα treatment on SAMHD1 protein levels in DCs.

### SAMHD1 levels in primary CD4^+^ T-lymphocytes are not upregulated by type I IFN treatment

**Figure 6:**
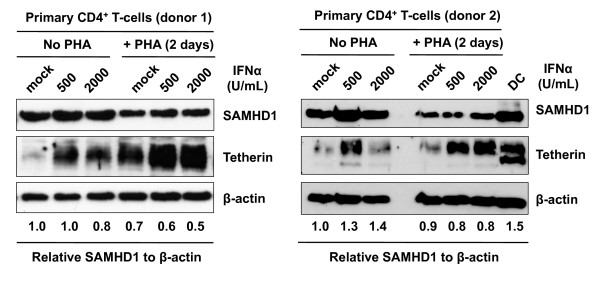
**SAMHD1 levels in primary CD4**^+ ^**T**-**lymphocytes are not upregulated by type I IFN treatment.** Primary CD4^+^ T cells from two healthy donors were treated with (+) or without PHA (5 μg/mL) for 48 hr and cultured in the presence of different amounts of IFNα for 24 hr. Whole cell lysates were subjected to immunoblotting for SAMHD1 and tetherin expression. β-actin was used as a loading control and relative levels of SAMHD1 compared to β-actin are shown. The data shown represents one of three independent experiments using cells from three different donors.

In addition to primary DCs, we analyzed SAMHD1 protein levels in resting and phytohemagglutinin (PHA)-activated primary CD4^+^ T-lymphocytes from two independent donors (Figure 6). We observed that resting and activated CD4^+^ T-lymphocytes expressed abundant SAMHD1, which was likely comparable to those in DCs (donor 2 results in Figure 6). These data are in agreement with the recent reports regarding SAMHD1 expression in resting and activated CD4^+^ T-lymphocytes [14,15]. Moreover, we observed that type I IFN treatment did not significantly affect SAMHD1 protein expression (Figure 6), indicating that endogenous SAMHD1 in primary CD4^+^ T-lymphocytes cannot be further upregulated by type I IFNs.

### Type I IFN treatment upregulates SAMHD1 protein expression in HEK 293T cells and HeLa cells

**Figure 7:**
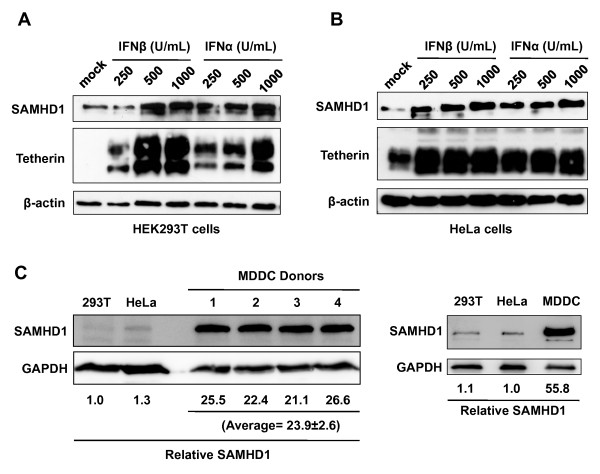
**Type I IFN treatment upregulates SAMHD1 expression in HEK 293T cells and HeLa cells.** HEK 293T cells (**A**) and HeLa cells (**B**) were treated with different amounts of IFNα or IFNβ as indicated for 24 hr and whole cell lysates were subjected to immunoblotting for SAMHD1 and tetherin expression. β-actin is shown as a loading control. (**C**) Comparison of endogenous SAMHD1 expression in HEK 293T cells, HeLa cells, and monocyte-derived DCs (MDDCs) from five independent donors. Relative SAMHD1 levels compared to GAPDH are shown.

As the levels of endogenous SAMHD1 protein in DCs and resting CD4^+^ T-lymphocytes were not affected by IFN treatment, we investigated the effect of type I IFN on SAMHD1 protein expression in HEK 293T and HeLa cell lines, which express relatively lower levels of endogenous SAMHD1 (Figure 7). Treatment of either cell line with IFNα or IFNβ significantly increased SAMHD1 protein levels compared to mock treated cells (Figure 7A and 7B, top panel). As expected, tetherin expression was also enhanced by type I IFN treatment (Figure 7A and 7B, middle panel), confirming that SAMHD1 is a type I IFN inducible protein in HEK 293T and HeLa cells. Comparison of endogenous SAMHD1 levels between HEK 293T cells, HeLa cells and DCs from five different donors revealed that DCs typically expressed 24- to 56-fold more SAMHD1 (Figure 7C). Together, these data suggest that SAMHD1 levels may only be upregulated by IFN treatment in cells that express low levels of endogenous SAMHD1 protein.

### Over-expression of SAMHD1 in HEK 293T cells or HeLa cells does not inhibit HIV-1 infection, but modestly decreases the intracellular dNTP pool in HeLa cells

**Figure 8:**
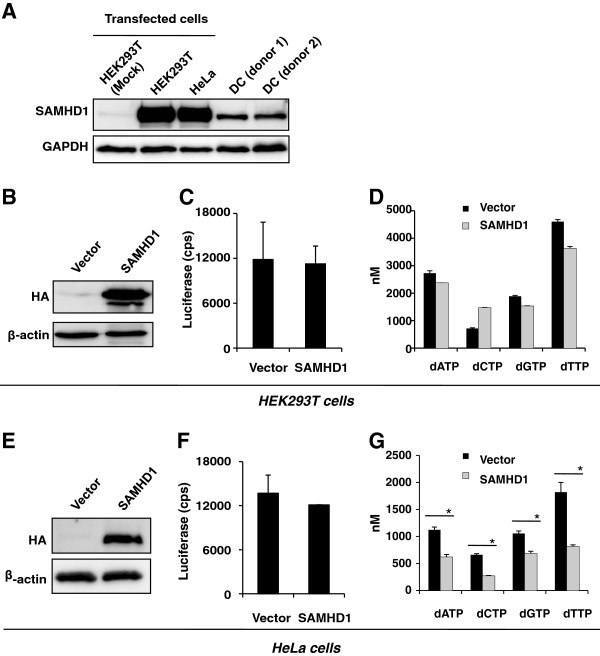
**Over-expression of SAMHD1 in HEK 293T cells or HeLa cells does not inhibit HIV-1 infection, but only modestly decreases the intracellular dNTP pool.** (**A**) HEK 293T and HeLa cells were transiently transfected with SAMHD1-expressing construct or an empty vector. SAMHD1 expression was detected by immunoblotting with SAMHD1-specific antibodies at 24 hr post-transfection. Equal amounts of total protein (20 μg) of the lysates of DCs from two donors and HEK 293T and HeLa cells were loaded. (**B**-**D**) HEK 293T cells and (**E**-**G**) HeLa cells were transiently transfected with HA-tagged SAMHD1-expressing construct or an empty vector. SAMHD1 expression in transfected HEK 293T cells (**B**) or HeLa cells (**E**) was detected by immunoblotting with anti-HA at 24 hr post-transfection. β-actin was used as a loading control. Transfected HEK 293T cells (**C**) or HeLa cells (**F**) were infected with HIV-Luc/VSV-G at an MOI of 0.5, and the infection was detected by measuring luciferase activity in the cell lysates at 48 hr post-infection and normalized to protein content (10 μg/sample). cps, counts per second. The intracellular dNTP pool of transfected HEK 293T cells (**D**) or HeLa cells (**G**) was measured at 24 hr post-transfection using the single-nucleotide incorporation assay. The asterisks indicate a significant difference (*p* <0.05) compared with the vector controls. The data shown represents one of two or three independent experiments. Error bars represent standard deviation of the mean of duplicate or triplicate samples.

Given that HEK 293T and HeLa cell lines are highly permissive to post-entry HIV-1 infection [33], and express lower SAMHD1 compared to primary DCs (Figure 7C), we investigated whether over-expression of SAMHD1 in these cells could cause an HIV-1-specific restriction phenotype and whether SAMHD1 retained its dNTP hydrolysis enzymatic activity. First, immunoblotting was performed to assess the relative levels of SAMHD1 overexpression in HEK 293T and HeLa cells compared to endogenous SAMHD1 in DCs. We found that both transfected HEK 293T and HeLa cells expressed high levels of SAMHD1 above the endogenous level observed for DCs from two different donors (Figure 8A).

We next determined the effect of SAMHD1 over-expression on the intracellular dNTP pool and VSV-G-pseudotyped single-cycle HIV-1 infection. SAMHD1 over-expression in HEK 293T and HeLa cells was confirmed by immunoblotting (Figure 8B and 8E). Transfected cells were either infected with VSV-G-pseudotyped HIV-1 and lysates measured for luciferase activity to determine HIV-1 infection or, cellular extracts were processed for dNTP quantification. Even in the presence of high SAMHD1 expression levels, there was no effect on HIV-1 infection in either HEK 293T cells or HeLa cells at 2 days post-infection (Figure 8C and 8F). Interestingly, SAMHD1 over-expression in HEK 293T cells had little effect on the dNTP concentration, at most a 1.3-fold decrease was observed for dTTP levels (Figure 8D). Comparatively, SAMHD1 over-expression in HeLa cells had a more pronounced effect on dNTP concentration and 1.8-, 2.4-, 1.5-, and 2.2-fold decreases were observed for dATP, dCTP, dGTP, and dTTP, respectively (*p* <0.05) (Figure 8G). These results indicate that SAMHD1 retains its enzymatic activity and is able to modestly hydrolyze dNTPs in HeLa cells; however, the effect is not significant enough to deplete the intracellular dNTP pool to a level that is capable of restricting HIV-1 infection.

## Discussion

In the current study, we examined the role of SAMHD1 in restricting HIV-1 infection of DCs and compared SAMHD1 expression levels following treatment with IFNs. Our results show that Vpx-mediated degradation of SAMHD1 in DCs can relieve a post-entry restriction block against HIV-1 by increasing the intracellular dNTP pool and promoting the accumulation of HIV-1 late reverse transcription products. However, early reverse transcription products were not affected by SAMHD1 degradation, consistent with previous findings in macrophages [12], suggesting that SAMHD1-mediated HIV-1 restriction mainly affects late reverse transcription. A previous study found that the HIV-1 genome is not completely reverse transcribed in quiescent lymphocytes, unlike in activated lymphocytes [41]. It is likely that HIV-1 early reverse transcription can be initiated in non-cycling cells with low dNTP concentrations, but the late reverse transcription cannot be completed. We also observed that there was no direct correlation between fold increase in HIV-1 late reverse transcription products and fold change in viral infectivity. A potential explanation for the difference is that HIV-1 late reverse transcription product levels may not fully reflect the efficiency of viral gene expression given the complexity of the viral life-cycle in DCs [33-35].

SAMHD1 functions as a dGTP-dependent phosphohydrolase [19,20], and its degradation with Vpx treatment in DCs increased accumulation of HIV-1 late reverse transcription products, suggesting that SAMHD1 regulates intracellular dNTP levels in DCs. We show that DCs contain low levels of dNTPs (~11–415 nM), within the range of resting T-lymphocytes (300–5,000 nM) [37], but below that in HIV-1 permissive cell types, such as activated peripheral blood mononuclear cells (PBMCs) (1.5–9.2 μM) [38] and activated CD4^+^ T-lymphocytes (3–30 μM) [37]. It appears that DCs have dNTP levels ~1.9- to 2.3-fold higher than macrophages (~50 nM) [16].

Although HIV-1 infection of DCs is enhanced in the presence of Vpx, the levels of p24 released into the supernatant from infected DCs are lower compared to those from macrophages and fully permissive target cells [32,33]. This suggests that SAMHD1 has a role in HIV-1 restriction in DCs, but it is likely that additional post-entry restriction steps exist to block HIV-1 infection in DCs. For example, APOBEC3A is highly expressed in myeloid-lineage cells and interacts with Vpx, leading to its degradation, which correlates with increased HIV-1 infection in primary monocytes [42]. Silencing of APOBEC3A relieves restriction of HIV-1 in macrophages, DCs and monocytes [43], and abolished deaminase activity of APOBEC3A in monocytes [44]. There also remains an unidentified cryptic sensor for HIV-1 infection in DCs dependent on newly synthesized viral capsid [29].

SAMHD1 restricts HIV1 infection in resting CD4^+^ T-lymphocytes by limiting reverse transcription through depleting intracellular dNTP concentrations [14]. Previous studies measuring dNTP levels in resting T-lymphocytes suggest that the intracellular dNTP pool is sufficiently low to restrict HIV-1 reverse transcription, which can be attributed to SAMHD1 activity [37,38]. Recent studies showed that T-cell activation does not significantly affect SAMHD1 expression in primary CD4^+^ T-cells treated with PHA or with anti-CD3/anti-CD28 for 2–3 days [14,15]. In agreement with these results, we observed that PHA-treatment of resting CD4^+^ T-cells for 2 days only slightly decreased SAMHD1 expression in activated CD4^+^ T-lymphocytes (by 10%- 30% in two donors, Figure 6). Activated CD4^+^ T-cells have a 3- to 8-fold higher dNTP concentration relative to resting CD4^+^ T-cells, while SAMHD1 expression remains the same in resting and activated CD4^+^ T-cells [14]. It is possible that intracellular dNTP levels can be significantly increased when CD4^+^ T-cells are activated and become dividing cells. How activated CD4^+^ T-cells upregulate the intracellular dNTP pool without decreasing SAMHD1 expression remains to be investigated.

We found that over-expression of SAMHD1 in dividing cell lines does not restrict HIV-1, similar to a study which found that SAMHD1 expression in dividing cell lines did not have an inhibitory effect on a range of viruses, including HIV-1 [45]. Our dNTP analysis in HeLa cells suggests that SAMHD1 is able to moderately deplete the dNTP pool; but the concentration of dNTPs was within the range of activated T-lymphocytes [37], suggesting dividing cell lines are capable of maintaining their dNTP pools in the presence of high levels of SAMHD1. It is possible that the catalytic activity of over-expressed SAMHD1 in HEK 293T and HeLa cells may be less stoichiometrically active than the endogenous protein in DCs, and/or that the transformed cell lines lack a potential cellular co-factor(s) for SAMHD1-mediated HIV-1 restriction function [46].

Analysis of SAMHD1 after IFN treatment indicated that neither type I nor type II IFN treatment affected SAMHD1 protein levels in DCs or primary CD4^+^ T-lymphocytes. However, analysis of *SAMHD1* mRNA levels at 6 hr post-treatment with IFN indicated a 2- to 4-fold increase in mRNA, suggesting that in DCs SAMHD1 is IFN sensitive, albeit transiently. Comprehensive analysis of the effect of IFNα treatment of DCs from 6 to 72 hrs indicated no change in SAMHD1 protein levels and a small transient increase in mRNA levels at 6 and 12 hr post-treatment. Furthermore, our data for IFN treatment of HEK 293T and HeLa cells, as well as previous studies [13] show that SAMHD1 is type I IFN inducible. Although Berger *et al*. observed increased SAMHD1 protein levels in primary monocytes upon IFNα treatment, we observed that primary monocytes express lower levels of SAMHD1 relative to DCs (data not shown), which could partially explain the difference in response to IFNα treatment across the two cell types. As we show that DCs have low levels of dNTPs, it is plausible that SAMHD1 expression and/or its activity is tightly regulated in these cells to ensure a minimal dNTP pool is maintained without causing detrimental effects on the cell, for example, DNA repair within cells requires carefully modulated dNTP levels [47,48]. It is also possible that post-transcriptional regulation of *SAMHD1* mRNA may affect SAMHD1 protein expression in the cell. A recent study identified naturally occurring splice variants of SAMHD1 [49], indicating that SAMHD1 expression and activity is regulated at a transcriptional level.

Interestingly, HIV-1 has no means of counteracting SAMHD1, and our recent study suggests that co-evolution of primate SAMHD1 and lentivirus Vpx led to the loss of the *vpx* gene in the HIV-1 precursor, SIVcpz, and consequently HIV-1 [50]. Additional studies also suggested that SAMHD1 restriction toward HIV-1 was evolutionarily maintained under positive selection and that antagonism of SAMHD1 by Vpx is species-specific [51,52], but that Vpx degradation of SAMHD1 was an acquired ability that arose through positive selection in lentiviruses [45]. We recently reported that common polymorphisms of *SAMHD1* are unlikely to contribute to the infection and natural control of HIV-1, at least in European and African-American individuals [53]. It is interesting to investigate whether polymorphisms of *SAMHD1* are associated with HIV-2 and SIV infections in humans and non-human primates, respectively.

SAMHD1 has been suggested to play a role in the innate immune responses to viral infections [54-58]. Our results indicate that SAMHD1 functions as an important restriction factor to counteract HIV-1 infection in DCs. Broader understanding of the mechanism of SAMHD1-mediated restriction in non-dividing cells and further investigation of the biological role of SAMHD1 is vital to enhancing our knowledge of HIV-1 infection and pathogenesis.

## Conclusions

Our results suggest that SAMHD1-mediated reduction of the intracellular dNTP pool in DCs is a common mechanism of HIV-1 restriction in myeloid cells. Endogenous expression of SAMHD1 in primary DCs or CD4^+^ T-lymphocytes is not further upregulated by type I IFNs.

## Methods

### Plasmids

HIV-1 proviral vector pNL-Luc-E^–^R^+^ contains a firefly luciferase reporter gene was a kind gift from Dr. Nathaniel Landau (New York University School of Medicine) [59]. HIV-1 proviral vector pNLAD8 (R5-tropic) was a kind gift from Dr. Eric Freed [60] (National Cancer Institute-Frederick). The SIV3+ plasmid, provided by Dr. Andrea Cimarelli [61], was used to produce SIVmac251 VLPs for delivery of Vpx into cells. A SIV3+ derivative, in which the *vpx* and *vpr* initiation codons were mutated, pSIVX-, was provided by Dr. Jacek Skowronski (Case Western Reserve School of Medicine) and has been described previously [12]. *Trans*-complemented Vpx-containing VLPs were produced using pCG.myc.Vpx that expresses Vpx from HIV-2_Rod_, and human SAMHD1 expression plasmid pCG-F-HA-SAMHD1 (kind gifts from Dr. Jacek Skowronski) or empty vector controls were used for transfections and over-expression in cell lines.

### Cell culture

Human PBMCs were isolated from the buffy coat of healthy blood donors as previously described [62]. Primary CD14^+^ monocytes and CD4^+^ T-lymphocytes were isolated from PBMCs by positive selection as described previously [36,62]. Immature DCs were generated from purified monocytes by treatment with granulocyte-macrophage colony-stimulating factor and IL-4 (50 ng/mL, R&D Systems) for 5 days as described previously [63,64]. Primary resting CD4^+^ T cells were cultured in the presence of 20 IU/mL of recombinant IL-2 (obtained from the NIH AIDS Research and Reference Reagent Program) and activated by 5 μg/mL of PHA (Sigma-Aldrich) for 2 days (short-term activation) as previously described [62]. DCs and CD4^+^ T-lymphocytes generated using these methods were more than 98% pure by flow cytometry analysis of surface markers as previously described [34,36,64]. Human embryonic kidney cell line HEK 293T cells, HeLa cells, and the HIV-1 indicator cell line GHOST/X4/R5 have been previously described [63,64].

### Generation of SIV VLP for DC transduction

SIV VLPs were generated by transfection of HEK293T cells with the appropriate plasmids, SIV3+ or SIV(X-), pCG.myc.Vpx and the VSV-G expressing vector pVSV-G as described [65]. Two days post-transfection, supernatants were harvested and filtered through a 0.45 μM filter and layered over a 25-45% sucrose step gradient. Gradients were ultra-centrifuged at 28,000 × g for 90 min at 4°C. Supernatants were collected from the gradient interface, diluted in PBS and ultra-centrifuged through a 25% sucrose cushion at 28,000 × g for 90 min at 4°C. VLPs were recovered in medium by gentle rocking at 4°C for 3 hr, aliquoted and stored at −80°C (protocol adapted from [65]).

### Treatment of DCs, CD4^+^ T-lymphocytes and cell lines with type 1 IFNs

Cells were treated with a range of concentration of IFNs or mock treated with medium, as indicated and described previously [32,34,35]. At 24 hr post-treatment, lysates were harvested and analyzed by SDS-PAGE and immunoblotting. All IFNs and other cytokines were purchased from PeproTech.

### Quantification PCR analysis of *SAMHD1* mRNA levels in IFN-treated DCs

Levels of *SAMHD1* mRNA levels in DCs treated with 2000 U/mL IFNα, IFNβ, or IFNγ for 6 or 24 hr were quantified by SYBR-green real-time quantitative PCR analysis using primer sets and protocols previously described [53]. Briefly, RNA from treated DCs at various time points was isolated using an RNeasy kit (QIAgen) and 200 ng of total RNA from IFN-treated DCs was used as input for cDNA synthesis, according to manufacturer’s instructions for SuperScript III First-Strand Synthesis System (Life Technologies).

### HIV-1 stocks

Single-cycle, luciferase reporter HIV-1 stock (HIV-Luc/VSV-G) was generated by calcium phosphate co-transfection of HEK 293T cells with the pNL-Luc-E^–^R^+^ and pVSV-G as described [33]. Replication-competent NLAD8 WT HIV-1 virus stocks were generated by transfection of HEK 293T cells with proviral vector pNLAD8 as described [62]. All virus stocks were harvested 2 days post-transfection and filtered through a 0.45 μM filter. The infectious units of virus stocks were evaluated by limiting dilution on GHOST/X4/R5 cells as described [64]. HIV-1 p24 concentrations of viral stocks were measured by ELISA (anti-p24-coated plates were purchased from the AIDS Vaccine Program, SAIC-Frederick, MD) as previously described [34].

### Transfections

HEK 293T cells were transfected using a calcium phosphate method to over-express SAMHD1 or vector controls, and cells were processed for downstream applications at 24 hr post-transfection. HeLa cells were transfected using the *Trans*IT-HeLaMONSTER transfection kit (Mirus) according to the manufacturer’s instructions. At 24 hr post-transfection, cells were processed for HIV-1 infection assays, dNTP analysis, or cell lysates were harvested for immunoblot analysis.

### Immunoblotting

Cells were harvested as indicated and lysed in cell lysis buffer (Cell Signaling) supplemented with protease inhibitor cocktail (Sigma-Aldrich). Cell lysates were subjected to 12% SDS-PAGE and immunoblotting as described [66]. Restore Western blot stripping buffer (Pierce) was used to strip antibodies from probed membranes. Super-signal chemiluminescence substrate (Pierce) was used to detect horseradish peroxidase-conjugated secondary antibodies. Polyclonal mouse antibody reactive to SAMHD1 (ab67820) was purchased from Abcam and used at 1 μg/mL in 5% milk in Tris-buffered-saline-Tween. Antibody to HLA-II was purchased from BD Biosciences. Tetherin antiserum was a kind gift from Dr. Paul Spearman (Emory University) and used as described previously [32] . Antibody specific to the HA-epitope (Ha.11 clone 16B12) was purchased from Covance. β-actin (Santa Cruz Biotechnologies) or GAPDH (AbD serotec) antibodies were used as loading controls. Immunoblotting images were captured using Molecular Imager ChemiDoc XRS instrument or Fujifilm Luminescent Image Analyzer (LAS 4000) and analyzed using Quantity One software (BioRad) or Multi Gauge V3.0 software (Fuji Film).

### HIV-1 infection assays

HIV-1 infection assays using luciferase reporter viruses were performed using a multiplicity of infection (MOI) range of 0.5-2 as described previously [64,67]. Cell lysates were obtained at the indicated times post-infection and analyzed for luciferase activity using a commercially available kit (Promega) according to the manufacturer’s instructions. Total cell protein was quantified using a BCA assay (Pierce) and all luciferase results were normalized to total protein content. For infection of DCs using replication-competent HIV-1, DCs (2.5×10^5^) were transduced with Vpx-containing VLPs for 2 hr and then incubated with HIV-1_NLAD8_ (20 ng p24-equivalent, MOI of 0.5) for 2 hr as described [32,34]. Cells were then washed thoroughly and cultured for the indicated times. Cell-free supernatants from the HIV-1-infected DCs were harvested for Gag p24 quantification by p24 ELISA at the indicated times post-infection. Whole cell lysates of HIV-1-infected DCs were subjected to immunoblotting for HIV-1 Gag detection as described [32].

### Quantitative PCR analysis of HIV-1 cDNA

Levels of early and late reverse transcription products in infected DCs were quantified by SYBR-green based real-time quantitative PCR analysis using primer sets previously described [33,34,68]. Briefly, 100 ng of genomic DNA from HIV-1 infected DCs were used as input for the detection of early or late reverse transcription products. All virus stocks were treated with DNaseI (40 U/ml; Ambion) prior to infections to avoid plasmid DNA contamination. DNA from infected cells at various time points was isolated using a DNeasy Blood and Tissue kit (QIAgen).

### Intracellular dNTP quantification of DCs

For dNTP analysis and quantification, cells were harvested and lysed in cold 65% aqueous methanol, heated to 95°C for 3 min and extracts dried in a speed vacuum. Reactions were prepared and analyzed as described previously [37]. Briefly, extracts were incubated with 200 fmol substrate, 50 nM HIV-1 reverse transcriptase (RT), 10 μM oligonucleotide dT, and RT reaction buffer. Reactions were incubated for 5 min at 37°C and terminated using 10 μl 40 mM EDTA, 99% formamide and heated at 95°C for 5 min. For analysis, reaction products were separated on a 14% polyacrylamide-urea denaturing gel (SequaGel, National Diagnostics) and analyzed on a PhosphorImager (PerkinElmer). Product extension was quantified by densitometry (Quantity One) and dNTP content was back calculated from percent product extended. VLP + Vpx (24 hr) reactions were initially diluted 1:3 to prevent substrate from being fully extended. The calculation of intracellular dNTP concentrations (nM) was based on the reported volumes of DCs [69], HEK 293T cells [70], and HeLa cells [71].

### Statistical analysis

Data were analyzed using a two-way ANOVA test and Student’s *T*-test and statistical significance was defined as *p* <0.05.

## Competing interests

The authors declare that they have no competing interests.

## Authors’ contributions

LW, CSG, SdS, and BK conceived the study, designed the experiments and participated in data analyses. CSG, SMA, SdS, CMC, HH, and JAH performed all the experiments and participated in the experimental design. CSG, SdS and LW wrote the manuscript. All authors read and approved the final manuscript.

## Supplementary Material

Additional file 1 **Table S1.** Intracellular dNTP levels in DCs treated with Vpx(+) or Vpx(-) VLPs. Click here for file
