# Supplementary material for: SAMHD1 restricts HIV-1 infection in dendritic cells (DCs) by dNTP depletion, but its expression in DCs and primary CD4+ T-lymphocytes cannot be upregulated by interferons
Source: Retrovirology. 2012 Dec 11;9:105. doi: 10.1186/1742-4690-9-105 (PMC3527137; doi:10.1186/1742-4690-9-105)
Supplement: Additional file 1 — Table S1. Intracellular dNTP levels in DCs treated with Vpx(+) or Vpx(-) VLPs. [file 1742-4690-9-105-S1.docx]

**Supplementary information**

**Table S1. Intracellular dNTP levels in DCs treated with Vpx(+) or Vpx(-) VLPs.**

| **Time** | **0 hr post-treatment** | | **12 hr post-treatment** | | **24 hr post-treatment** | |
| --- | --- | --- | --- | --- | --- | --- |
| **VLPs** | **Vpx(-)** | **Vpx(+)** | **Vpx(-)** | **Vpx(+)** | **Vpx(-)** | **Vpx(+)** |
| **Donor #1** |  |  |  |  |  |  |
| dATP (nM) | 95.1±9.9 | 126.6±23.6 | 87.8±3.0 | 119.8±0.6 | 82.2±9.0 | 609.2±193.4 |
| dCTP (nM) | 414.9±21.8 | 466.6±19.0 | 108.0±13.1 | 122.1±9.4 | 87.4±18.1 | 241.0±21.9 |
| dGTP (nM) | 152.8±2.2 | 156.4±69.3 | 95.4±11.7 | 121.2±2.8 | 85.5±16.3 | 232.4±37.7 |
| dTTP (nM) | 147.2±1.8 | 122.5±2.9 | 18.7±3.2 | 28.7±8.6 | 11.4±3.3 | 96.0±6.5 |
| **Donor #2** |  |  |  |  |  |  |
| dATP (nM) | 77.6±1.8 | 103.8±4.3 | 85.8±1.5 | 321.4±58.5 | 68.5±3.3 | 1384.0±246.6 |
| dCTP (nM) | 188.3±17.3 | 208.3±43.2 | 72.8±10.0 | 202.8±3.8 | 52.4±18.4 | 364.7±47.8 |
| dGTP (nM) | 203.3±0.1 | 206.1±6.8 | 145.1±13.3 | 534.4±33.8 | 153.4±76.1 | 1089.1±213.9 |
| dTTP (nM) | 71.5±9.7 | 88.0±0.0 | 33.6±1.7 | 164.9±27.6 | 25.1±4.6 | 363.5±49.0 |

The numbers represent the mean values ± S.D. derived from the quantification of the extension products in the HIV-1 RT-based single nucleotide primer extension assay (refer to Fig. 2B).
